# A New Method for Preparing Cross-Sections of Polymer Composite Membranes for TEM Characterization by Substrate Stripping and Double-Orientation Embedding

**DOI:** 10.3390/membranes15100288

**Published:** 2025-09-24

**Authors:** Hongyun Ren, Zixing Zhang, Yi Li, Shulan Liu, Xian Zhang

**Affiliations:** 1 Center of Analytical Instrument, Institute of Urban Environment, Chinese Academy of Sciences, Xiamen 361021, China; hyren@iue.ac.cn (H.R.);; 2 National Key Laboratory of Regional and Urban Ecological Safety, Institute of Urban Environment, Chinese Academy of Sciences, Xiamen 361021, China; 3 School of Chemical Engineering and Technology, Sun Yat-sen University, Zhuhai 519082, China

**Keywords:** polymer composite membrane, transmission electron microscopy, ultra-thin section of membrane cross-section, double-orientation embedding, embedding device

## Abstract

Membrane technology plays a vital role in environmental protection, chemical industry, and pharmaceuticals, where understanding the “structure-property” relationship of composite membranes through transmission electron microscopy (TEM) is crucial. Conventional ultramicrotomy methods for preparing ultra-thin sections of polymer composite membranes often result in significant damage and non-uniform thickness due to interference from non-woven substrates. In this study, we developed an innovative substrate stripping and double-orientation embedding technique that overcomes these limitations. A special embedding device was designed to facilitate the preparation of polymeric membrane cross-sections for TEM analysis. The device incorporates dual functionality, enabling both non-woven substrate detachment and bidirectional alignment of functional membrane layers. TEM characterization showed that the ultra-thin sections of membrane cross-sections prepared using the improved method were damage-free (0% damage rate), had uniform thickness, and showed distinct structural clarity. This method addressed three major challenges: (i) substrate-induced section damage, (ii) orientation deviation, and (iii) interlayer separation. This advancement provides researchers with a reliable tool for accurate cross-sectional analysis of composite membranes, facilitating deeper insights into membrane microstructure-performance relationships.

## 1. Introduction

Over the past few decades, membrane technology has evolved into one of the key technologies in the field of gas and liquid separations [1]. It plays a crucial role in diverse applications, including seawater desalination, wastewater treatment, pharmaceutical production, and food processing [2,3]. Membranes are typically classified into inorganic membranes and polymeric membranes [4]. Among these, polymeric membranes dominate current market applications due to their high flexibility, easy preparation, and lower cost [5]. Polymeric membrane materials mainly include polyethylene (PE), polypropylene (PP), polysulfone (PSf), polyethersulfone (PES), polyamide (PA), and cellulose acetate (CA) [6,7]. Composite membranes typically consist of a non-woven substrate, a porous intermediate layer, and a top selective layer [8,9]. The non-woven substrate and intermediate layer must possess excellent chemical and thermal stability, suitable mechanical strength, and facilitate full-scale manufacturing [10,11]. The selective layer exhibits outstanding selectivity and high solvent permeability [8,12]. Based on the requirements for high selectivity, permeability, hydrophilicity, thermal stability, mechanical strength, and antimicrobial properties of polymeric membranes, composite membranes are usually optimized by modifying material, additive, membrane structure, and top selective layer [13,14,15].

TEM is one of the most important characterization techniques for studying the “structure-property” characteristics of composite membranes [16]. Since membrane performance is closely related to its structure, an in-depth understanding of the microstructure of composite membranes is of great significance for interpreting their performance and optimizing preparation processes [17]. TEM can characterize the internal nano-/micro- structures of membranes, including pore structure, pore size distribution, layer thickness, interfacial morphology, and modified nanomaterials [18,19,20]. Samples suitable for TEM observation must be electron-beam transparent thin films with a thickness ranging from tens to hundreds of nanometers [21]. Composite membranes typically need to be cut into ultra-thin sections with a thickness of approximately 100 nm. In membrane modification research, the thickness of the selected or modified layers is one of the most critical parameters, which may range from tens to hundreds of nanometers [22,23,24]. To accurately analyze the hierarchical structure and thickness of the membrane, the sectioning direction should be perpendicular to the membrane’s extension direction.

**Figure 1:**
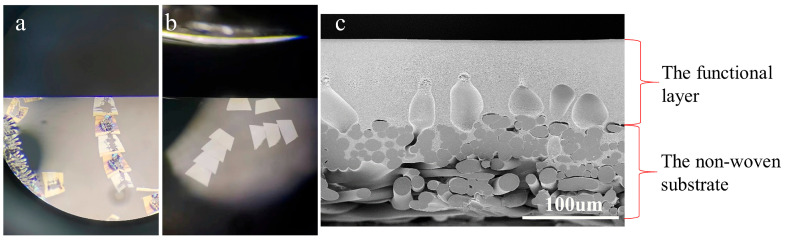
(**a**) The damaged section and (**b**) the normal section floating on the liquid surface of the knife trough. (**c**) The cross-section of the composite membrane under the scanning electron microscope.

Currently, the primary approaches for preparing ultra-thin sections of polymeric membranes involve room-temperature ultramicrotomy and cryoultramicrotomy [16,25,26]. For room-temperature ultramicrotomy, directly embedding the membrane samples in resin resulted in ultra-thin sections with uneven thickness and significant damage (Figure 1a). Polymer composite membranes typically contain a non-woven substrate formed by pressing crisscrossed PE/polyester (PET) fibers [8,9], as shown in Figure 1c. Due to distinct differences in hardness, density, and toughness between the fibrous substrate and the surrounding embedding block, the sectioning knife experienced uneven force [27]. Furthermore, the extreme thinness of the sections (approximately 100 nm) exacerbated section damage. Although some areas of the damaged sections could still be observed under high magnification, the scarcity of intact areas posed significant challenges for TEM characterization. Similarly, cryoultramicrotomy for preparing sections of composite membranes with non-woven substrates not only suffered from section damage but also tended to cause delamination, such as the separation of the top selective layer from the base membrane. This limitation arises because cryoultramicrotomy typically employs sucrose solution as the embedding medium, which provides weaker adhesion and fixation to polymers compared to cured resins. As a result, delamination is more prone to occur in composite membranes with loosely bonded interlayers. Thus, the preparation of ultra-thin sections of composite membranes via conventional methods remains challenging, which needs to be improved to enhance the cross-sectional sample quality in TEM characterization.

In the TEM characterization of composite membranes, the structure and morphology of the functional layer constitute the primary focus of research. Therefore, it is feasible to embed the membrane after removing its non-woven substrate to prepare cross-sectional samples of the functional layer. Although the fibrous substrates of some composite membranes can be directly peeled off [28], the functional layers—characterized by their extreme thinness and low density—are prone to bending and tilting under the influence of thermal effects during resin embedding and polymerization. This makes it difficult to ensure that the membrane’s extension direction is precisely perpendicular to the sectioning direction during ultra-thin section preparation. Furthermore, the functional layers of some membranes are thin, brittle, or tightly bonded to the substrates, rendering intact sheet separation challenging. Zhou et al. demonstrated that free-standing PA membranes could be obtained by dissolving the PSf support layer in dimethylformamide from thin-film composite membranes [29]. Nevertheless, such free-standing PA layers are exceedingly thin and fragile, making them highly challenging to handle directly for the preparation of cross-sectional samples suitable for TEM analysis—consistent with the limitations discussed earlier. To overcome these limitations, this study developed a method for stripping the non-woven substrate based on resin embedding technology and designed a specialized embedding device. This method involves a two-step resin embedding process. During the initial embedding step, the functional layer of the membrane is embedded and cured in situ, while the fibrous layer is removed as an intact sheet. The second embedding step ensures complete resin impregnation of the functional layer and orients it perpendicular to the sectioning direction, thereby facilitating subsequent ultramicrotomy preparation. In this research, ten types of polymeric membranes were selected, and their cross-sectional sections were prepared using both the direct resin-embedding method and the improved sample preparation method. The effectiveness of these two methods was compared through TEM characterization and energy dispersive spectroscopy (EDS) analysis. The proposed method significantly enhanced the structural integrity of critical regions in the samples, providing a solid foundation for nanoscale high-resolution microstructural characterization and accurate elemental distribution analysis. Additionally, for membrane samples lacking a non-woven substrate but exhibiting thinness, low density, and difficulty maintaining orientation during embedding, they can be floated on a liquid surface, transferred onto a base membrane, and subsequently processed using this technique. The purpose of this study is to address critical limitations inherent in traditional sample preparation techniques and to offer a robust and high-quality sample pretreatment solution for advancing membrane science and technology.

## 2. Materials and Methods

### 2.1. Materials

Ultrafiltration membranes (UF), nanofiltration membranes (NF), and reverse osmosis membranes (RO) were investigated in this work. UF-1, UF-2, and UF-3 were kindly provided by the Membrane Science and Technology Research Group at the Institute of Urban Environment, Chinese Academy of Sciences (Xiamen, China). NF-1 was kindly provided by the Clean Energy Technology and Carbon Materials Research Group at the same institute (Xiamen, China). NF-2 was donated by the Institute of Energy Research, Jiangxi Academy of Sciences (Nanchang, China), while NF-3 and NF-4 were provided by the School of Chemical Engineering and Technology, Sun Yat-sen University (Zhuhai, China). RO-1 was obtained from Sunrain Group Co., Ltd. (Lianyungang, China), and RO-2 was acquired from Wanhua Chemical Group Co., Ltd. (Yantai, China). UF-1, UF-2, and UF-3 consist of a PES functional layer and a non-woven substrate. UF-2 and UF-3 were doped with low and high concentrations of molybdenum disulfide (MoS_2_) nanosheets, respectively. NF-1 is composed of a PA selective layer, a PSf ultrafiltration intermediate layer, and a non-woven substrate. NF-2, NF-3, and NF-4 comprise a PA functional layer and a non-woven substrate. The selective layer of NF-4 was doped with zinc-based zeolitic imidazolate framework (ZIF-8) nanoparticles via in situ interfacial polymerization. RO-1, RO-2, and RO-3 are structured with a PA functional layer, a porous PSf sublayer, and a non-woven substrate. The embedding medium used was Spurr’s resin, purchased from Electron Microscopy Sciences, LLC (Hatfield, PA, USA). Ultra-thin sections were prepared using glass knives produced by Leica Microsystems Inc. (Wetzlar, Germany).

### 2.2. Experimental Methods

#### 2.2.1. Direct Resin Embedding Method

The control samples (UF-1, UF-2, UF-3, NF-1, NF-2, NF-3, NF-4, RO-1, RO-2, RO-3) were prepared using a conventional ultramicrotomy method. The detailed procedures are as follows:

(1) Sample preparation. Firstly, membrane samples were cut into strips measuring 5 mm × 0.5 mm. Then, each strip was gently folded into an “L” shape and heated in an oven at 40 °C for 2 h.
(2) Resin Impregnation and Embedding. The folded samples were placed into embedding molds and immersed in resin. Samples were soaked in the resin for 12 h to ensure complete penetration.
(3) Orientation and Polymerization. The long arm of the “L” shape was adjusted to be parallel to the long axis of the embedding slot, and the short arm parallel to the short axis. The resin was polymerized in an oven at 70 °C for 24 h.
(4) Sectioning. The embedded samples were trimmed to expose the target region, followed by preparation of 100 nm-thick sections using an ultramicrotome (UC7, Leica, Wetzlar, Germany). Finally, sections were collected on copper grids and allowed to air-dry.

#### 2.2.2. Substrate Stripping Technique and Double-Orientation Embedding Method

**Figure 2:**
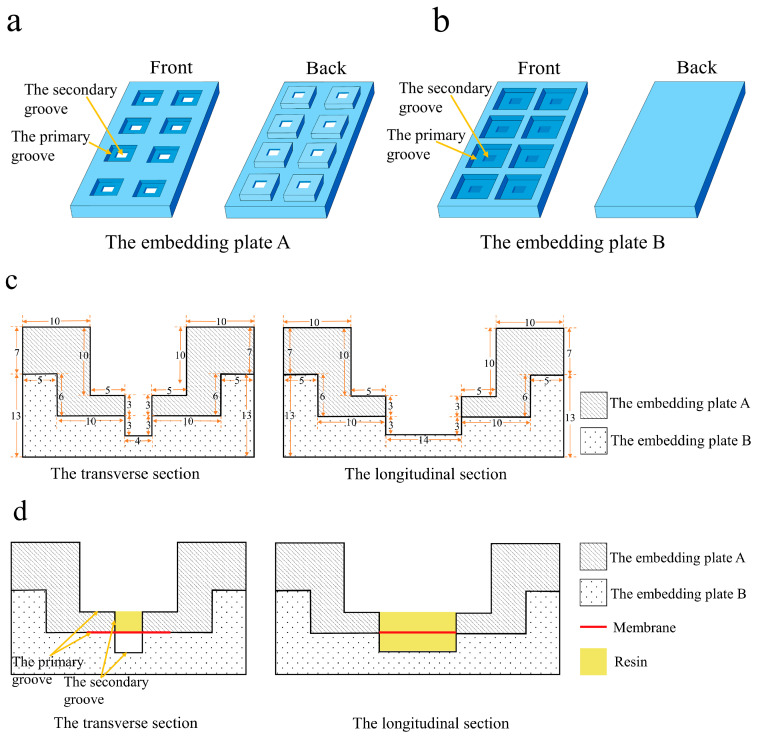
Structural diagrams of the embedding device. (**a**) The embedding plate A. (**b**) The embedding plate B. (**c**) The cross-sectional dimensions of a single embedding slot formed by the combination of embedding plate A and embedding plate B. Units are in millimeters. (**d**) Cross-sectional view of a single embedding slot after combining plates A and B. The transverse section shows the positional relationships among the embedding device, membrane, and resin during the first embedding process. The longitudinal section illustrates the positional relationships during the second embedding process.

**Figure 3:**
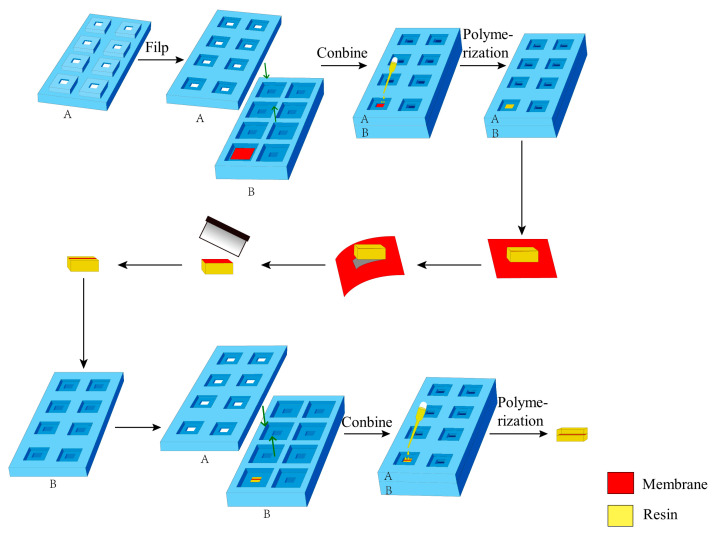
Schematic diagram illustrating the process of preparing the cross-sectional ultra-thin sections of composite membranes. It shows the steps from initially placing the membrane (with functional layer facing up) in the primary groove of the embedding plate B, combining it with the plate A, impregnating with resin in the secondary groove of the plate A, conducting the first resin embedding and polymerization, removing the fiber substrate, trimming and marking the membrane, to finally performing secondary embedding and polymerization. This process ensures that the non-woven fabric substrate of the membrane is removed and the membrane is directionally embedded, thereby facilitating the preparation of high-quality ultra-thin sections.

The embedding device was fabricated from silicone rubber, which is resistant to high temperatures and facilitates easy demolding. It consists of two parts: embedding plate A and embedding plate B, as shown in Figure 2. The embedding area of each part is equipped with a primary groove and a secondary groove, respectively. The two embedding plates are assembled via the primary grooves. Before the first embedding, the membrane was positioned in the primary groove of the embedding plate B. Then, it was combined with the embedding plate A to secure the membrane in a straight state. A clamping device was used to ensure the two embedding plates closely fitted. During the first embedding, liquid resin was added into the secondary groove of the embedding plate A on the membrane. The resin diffused slightly into the membrane without leaking. Prior to the second embedding, the prepared embedding block was inverted and positioned into the secondary groove of the embedding plate B, with the functional layer of the membrane parallel to the bottom of the embedding plate. Then, resin was added to the secondary groove of the embedding plate A for embedding. Ultimately, the membrane was embedded and immobilized within the center of a cuboid embedding block (Figure 3), which is approximately 4 mm × 6 mm × 14 mm in size.

**Figure 4:**
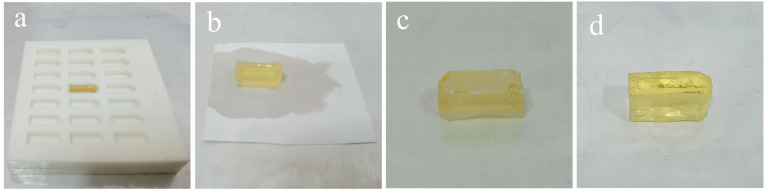
The physical objects of the self-made simple embedding device and embedding blocks. (**a**) The first embedding. (**b**) The embedding block prepared by the first embedding. (**c**) The embedding block of the membrane functional layer after stripping off the non-woven substrate. (**d**) The embedding block prepared by the second embedding.

The cross-sectional ultra-thin sections of composite membranes (UF-1, UF-2, UF-3, NF-1, NF-2, NF-3, NF-4, RO-1, RO-2, RO-3) were prepared using the improved method with the self-made embedding device. The procedure involved in situ resin impregnation of the functional layer side, followed by fiber layer separation after polymerization, trimming, marking, and secondary directional embedding (Figure 3). The specific procedures are as follows:

(1) Sample preparation. The membrane was cut into a rectangle approximately 2.5 cm on each side and heated in an oven at 40 °C for 2 h. Subsequently, the membrane was positioned in the primary groove of the embedding plate B with the functional layer facing upward. Embedding plate A was assembled with plate B, and the assembled plates were tightly clamped around the edges using a fixing device.
(2) Resin Impregnation. 120 µL of epoxy resin was added to the sample in the secondary groove of embedding plate A, followed by impregnation for 1 h. Then, an additional 120 µL of epoxy resin was added to the sample in the same groove, extending impregnation to 12 h.
(3) First Resin Embedding and Polymerization. The secondary groove of the embedding plate A (approximately 3 mm in depth) was filled with epoxy resin, as shown in Figure 4a. Polymerization was conducted in an oven at 70 °C for 24 h.
(4) Fiber Substrate Removal. The embedding plates A and B were disassembled to remove the embedding block (Figure 4b). Subsequently, the embedding block was clamped with tweezers, and the membrane adjacent to the block was manually peeled off. The functional layer of the membrane remained adhered to the bottom of the embedding block, as shown in Figure 4c.
(5) Membrane Trimming and Marking. The lateral regions of the membrane functional layer were trimmed, preserving only a 1–2 mm wide central portion. Charcoal pencil marks were lightly applied to both sides of the membrane to facilitate sample position identification during trimming and sectioning.
(6) Secondary Embedding. The embedding block was positioned in the secondary groove of the embedding plate B (membrane side upward), and plate A was assembled. After securing the assembly, the secondary groove of plate A was filled with resin and impregnated for 4 h.
(7) Second Polymerization. The oven temperature was set to 70 °C for another 24 h polymerization.
(8) Sectioning. The embedded block was trimmed. Then, ultra-thin sections were cut using an ultramicrotome, collected on copper grids, and air-dried.

#### 2.2.3. TEM Characterization and EDS Analysis

The cross-sections of composite membranes (UF-1, UF-2, NF-1, NF-2, NF-3, NF-4, RO-1, RO-2) prepared by both methods were characterized using a 120 kV transmission electron microscope (TEM, H-7650, Hitachi, Tokyo, Japan). The elemental mapping of the cross-sections of theUF-3, NF-4, and RO-3 membranes was studied using energy dispersive spectroscopy (EDS, XFlash 6T-30, Bruker, Ettlingen, Germany) coupled to a 200 kV transmission electron microscope (Talos F200i, Thermo Scientific, Waltham, MA, USA).

## 3. Results and Discussion

### 3.1. Cross-Sections of Membranes Prepared by Direct Resin-Embedding Method

**Figure 5:**
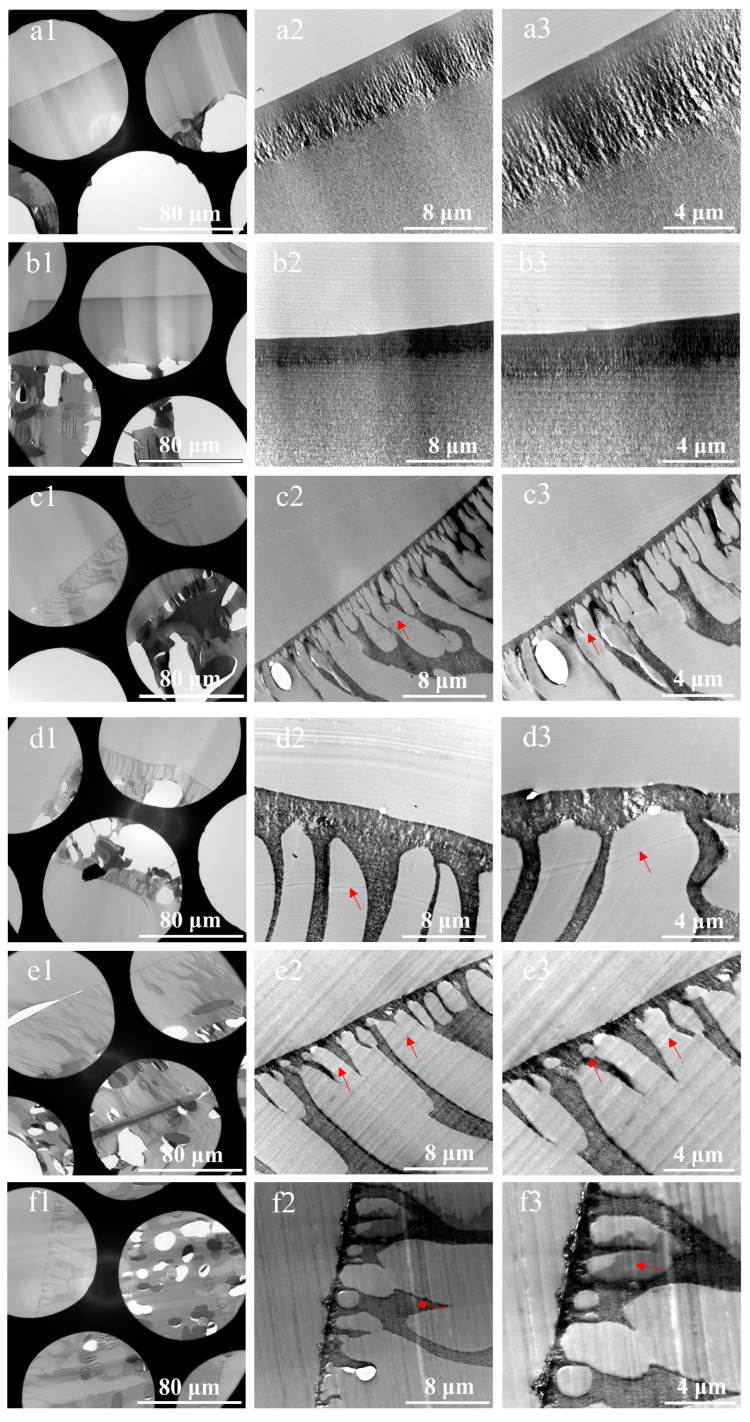

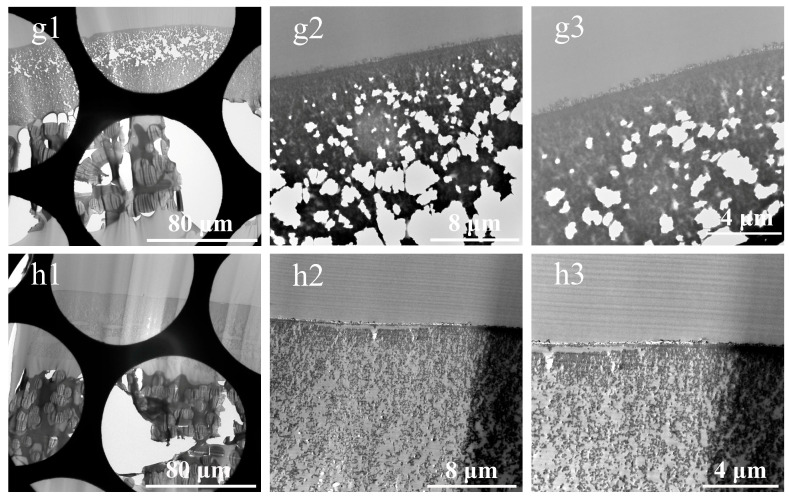
Cross-sectional TEM images of ultra-thin sections of composite membrane prepared using direct embedding method, (**a1**–**a3**) UF-1, (**b1**–**b3**) UF-2, (**c1**–**c3**) NF-1, (**d1**–**d3**) NF-2, (**e1**–**e3**) NF-3, (**f1**–**f3**) NF-4, (**g1**–**g3**) RO-1, (**h1**–**h3**) RO-2. The red arrows indicate the knife marks.

Composite membranes, owing to their porous structure with pore sizes typically above 0.1 nm [30,31], exhibit favorable permeability to the low-viscosity embedding medium Spurr’s resin (7.8 CPS). Therefore, the composite membranes can be directly impregnated with pure epoxy resin without ethanol transition. Eight types of composite membranes were selected, trimmed into strips, and impregnated together with their non-woven substrates. The TEM characterization results of ultra-thin sections of membrane cross-sections prepared using this method are shown in Figure 5. The results reveal five major issues in the ultra-thin sections:

(i.) Severe damage to the sections. This issue stems from the fact that the non-woven layer, composed of compressed PE/PET fibers with random orientations, possesses high density and negligible internal porosity, severely hindering resin penetration. The resulting embedding regions exhibited non-uniform properties, showing significant differences in hardness, toughness, and density. Consequently, the sections were prone to fragmentation, rendering the preparation of intact sections challenging. Even in partially preserved regions, localized deformation of the membrane functional layer was observed, as illustrated in Figure 5 (d1,g1).
(ii.) Non-uniform thickness of functional layers. Owing to the thickness–mass contrast principle in TEM, when an electron beam passes through a sample, a fraction of the electrons undergo scattering [32]. The sample thickness exhibits a positive correlation with the number of scattered electrons, which in turn influences the brightness (grayscale) of the TEM image. This grayscale variation can be used to intuitively assess local variations in sample thickness. Therefore, in our study, the uniformity of the membrane was evaluated by analyzing the grayscale distribution in cross-sectional TEM images. As shown in Figure 5(a1–c1,h1), alternating dark and bright bands parallel to the knife edge are visible on the sections, corresponding to regions of greater and lesser thickness, respectively. This phenomenon was attributed to the vibration between the knife and the sample, induced by the poor cutting performance of embedding blocks resulting from the density and hardness of the non-woven substrates.
(iii.) Presence of voids within functional layers, as evidenced in Figure 5(a1–a3,c1–h3). On the one hand, the presence of the fiber layers increased overall membrane thickness, exacerbating the difficulty of resin impregnation and causing voids due to insufficient penetration. On the other hand, uneven section thickness rendered excessively thin areas prone to void formation.
(iv.) Pronounced knife marks in partial sections, as displayed in Figure 5(c2,c3,d2,d3,e2,e3,f2,f3). The red arrows indicate the knife marks. Besides the possible presence of hard particles within the samples, small notches on the knife edge occurred when sectioning harder or thicker regions, resulting in knife marks. These artifacts compromised the thickness uniformity of the sections and obscured membrane structures.
(v.) Sample orientation deviation. During embedding using this method, the samples were directly immersed in resin without physical fixation. During subsequent processing, the sample positions were prone to displacement due to temperature variations and resin polymerization shrinkage. This misalignment led to deviations in sample orientation. Consequently, precise perpendicular alignment of the ultramicrotome knife relative to the membrane’s extension direction was unattainable. Therefore, the thickness of membrane layers determined by TEM deviated from the actual values.

To conclude, direct embedding of composite membranes poses significant challenges for obtaining high-quality ultra-thin section specimens.

### 3.2. Cross-Sections of Membranes Prepared by Substrate Stripping and Double-Orientation Embedding Technique

According to the above results, it is necessary to remove the non-woven substrate of composite membranes to improve the quality of cross-sectional samples prepared for TEM characterization. Here, a substrate stripping and double-orientation embedding technique was used to prepare the cross-sectional membrane samples. In this method, the functional layer of composite membranes was in situ cured with epoxy resin to facilitate substrate stripping. The epoxy resin infiltrated into the membrane from the functional layer side. On the one hand, the thin and fragile polymer functional layer was fixed in situ, preventing damage during separation from the substrate. On the other hand, the hardness and toughness of the functional layer were significantly enhanced, enabling facile separation. Additionally, the fibers within the non-woven substrate constitute a continuous and dense structure, which inherently resists fracture without mechanical intervention. Therefore, following partial embedding and curing on the functional layer side, the layers could be readily separated. To address the tendency of most composite membranes to curl due to the non-woven substrate, the membrane was placed and secured flat within the primary groove during the initial processing step. Furthermore, the interlocking of the two embedding plates via the primary groove ensured precise alignment of the secondary grooves. In step two, resin was added incrementally in two aliquots to mitigate significant diffusion in highly permeable membranes, which could otherwise lead to insufficient impregnation. This issue can be mitigated by increasing resin addition cycles. Moreover, the membrane functional layer became translucent or transparent after polymerization, necessitating marking of the membrane edges with a charcoal pencil to facilitate sample positioning during block trimming and sectioning. The charcoal pencil was chosen for its non-invasive nature, preventing penetration into the sample interior. Prior to sectioning, the marked regions on the embedding block were trimmed away.

**Figure 6:**
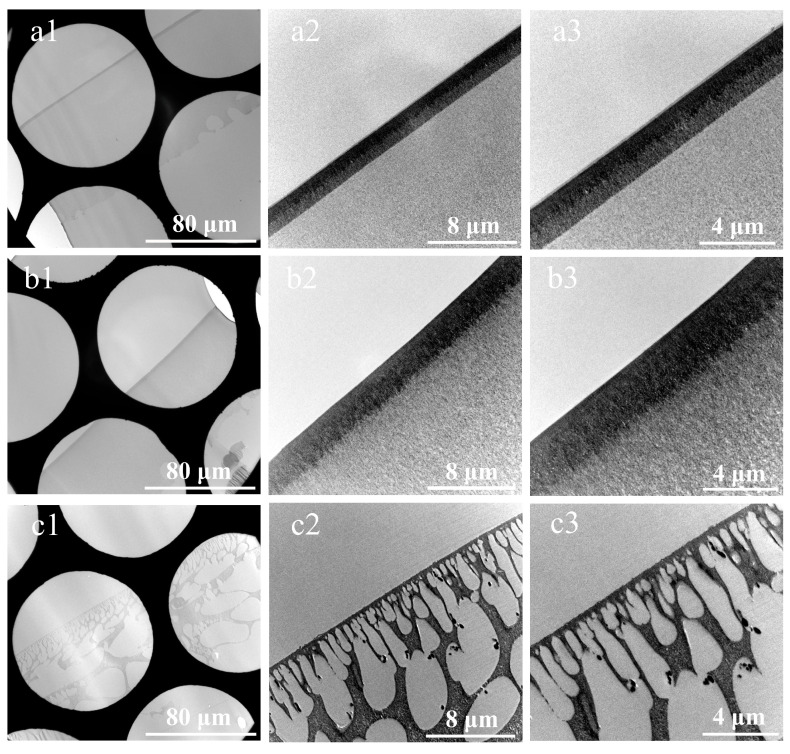

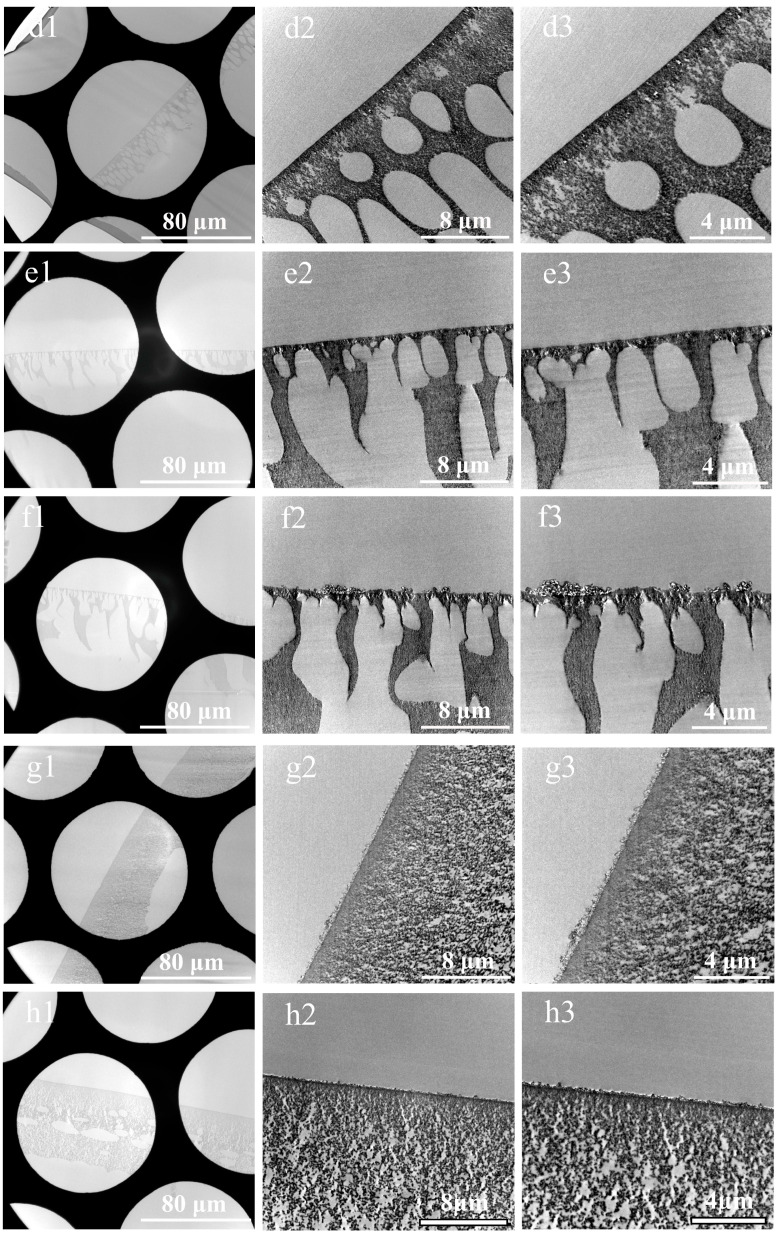
Cross-sectional TEM images of ultra-thin sections of composite membranes prepared by the substrate stripping and double-orientation embedding technique. (**a1**–**a3**) UF-1, (**b1**–**b3**) UF-2, (**c1**–**c3**) NF-1, (**d1**–**d3**) NF-2, (**e1**–**e3**) NF-3, (**f1**–**f3**) NF-4, (**g1**–**g3**) RO-1, and (**h1**–**h3**) RO-2.

Following drying, the prepared cross-sectional samples were characterized using the TEM. Figure 6 shows the eight composite membranes prepared using the double-embedding method, respectively. Compared to Figure 5, the membranes UF-1 and UF-2 exhibit superior impregnation by the embedding medium, with no voids or folds observed in the sections. In addition, the PES skin layers in Figure 6(a1–a3,b1–b3) show distinct boundaries, facilitating accurate thickness measurement. In Figure 6(c1–c3), the membrane NF-1 displays no knife marks or voids, with a clear structural morphology. As shown in Figure 6(d1–f3), the functional layers of membranes NF-2, NF-3, and NF-4 were relatively thin, measuring approximately 20–40 µm. These layers were fully impregnated with resin, uniformly polymerized, and structurally well-preserved. In Figure 6(f1–f3), the selective layer of the thin-film nanocomposite membrane modified with ZIF-8 nanomaterials formed protrusions on the membrane surface. The dimensions, structures, and binding sites of these protrusions were clearer than those in Figure 5(f1–f3). Figure 6(g1–g3) shows that the PSf sublayer of the membrane RO-1 was fully impregnated with resin, and the prepared section was continuous without voids. From Figure 6(h1–h3), it could be seen that the brightness of the membrane RO-2 section was consistent, indicating uniform thickness.

In summary, the eight cross-sectional sections of composite membranes prepared using the improved method exhibited no voids, minimal knife marks, uniform thickness, and clear structural features, which significantly outperformed those prepared via direct embedding. Furthermore, for some composite membranes with soft polymer matrices, the microstructure of the cross-sections prepared using room-temperature ultramicrotomy is prone to deformation, leading to artifacts. For such membranes, hardening treatment with the chemical stain osmium tetroxide can be applied before embedding using this method. Alternatively, after resin embedding and polymerization via this method, sectioning can be performed at low temperatures using a cryoultramicrotome [33].

The incorporation of a wide range of nanomaterials into distinct regions of thin-film nanocomposite membranes has been a key strategy for enhancing their performance, particularly in terms of permeability and selectivity [34,35,36]. The specific location of nanomaterial incorporation significantly influences membrane functionality. Accurate delineation of the actual spatial distribution and positional characteristics of nanofillers is a critical prerequisite for deriving valid conclusions. Consequently, more precise characterization methods should be exploited to clearly illustrate such information, so as to further elucidate the mechanisms underlying the effects of nanomaterials on membranes [37]. In this study, the cross-sections of three distinct membranes—an UF membrane (UF-3) incorporated with MoS_2_ nanosheets, a NF membrane (NF-4) whose selective layer was modified with ZIF-8 nanomaterials via in situ interfacial polymerization, and a RO membrane (RO-3) fouled by saline wastewater from a sewage treatment plant—were prepared using both the improved method and the direct resin-embedding method for elemental mapping analysis via EDS.

**Figure 7:**
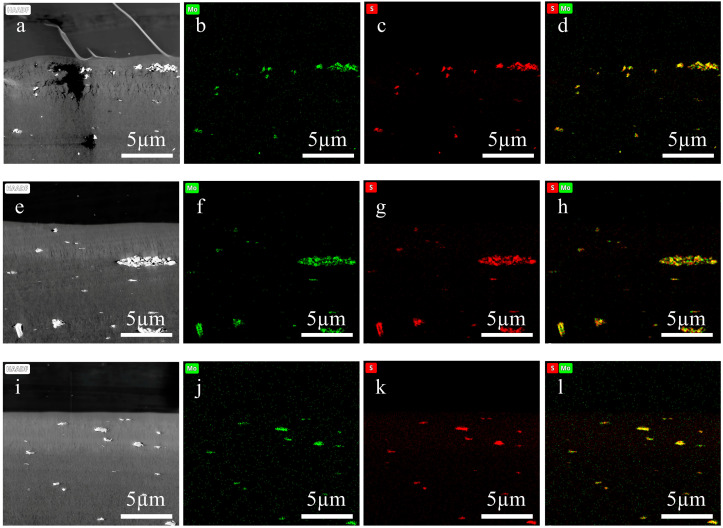
Elemental mappings of the cross-sectional ultra-thin sections of UF-3. (**a**–**d**) Sections prepared using the direct embedding method. (**e**–**l**) Sections prepared by the substrate stripping and double-orientation embedding technique. (**b**,**f**,**j**) Mo elemental maps, (**c**,**g**,**k**) S elemental maps, (**d**,**h**,**l**) overlay maps of Mo and S.

As shown in Figure 7a–d, Figures S1a–e and S2a–e, the cross-sectional samples of the three membranes prepared by the direct resin-embedding method all exhibited significant damage and deformation. These structural defects compromised sample integrity and severely undermined the accuracy of subsequent EDS analysis. In the UF-3 membrane, the loss of regions potentially enriched with MoS_2_ nanosheets resulted in incomplete elemental distribution data. For the NF-4 membrane, sectioning damage led to artificial displacement and aggregation of ZIF-8 nanoparticles within the PA selective layer, preventing an accurate representation of their actual doping state. In the RO-3 membrane, structural imperfections hindered precise evaluation of foulant deposition morphology and interfacial adhesion. Furthermore, even in preserved areas, physical deformations such as wrinkling, compression, or tearing could displace target elements in the elemental maps, potentially leading to misinterpretation of the spatial distribution of nanosheets or foulants.

In contrast, as depicted in Figure 7e–l, Figures S1f–j and S2f–j, the cross-sectional samples of all three membranes prepared using the substrate stripping and double-orientation embedding technique exhibited high structural integrity, with smooth surfaces free from deformation or damage. This method provided a robust foundation for acquiring accurate submicron-scale elemental mapping data. For the UF-3 membrane, EDS mapping clearly revealed characteristic distribution patterns of molybdenum and sulfur with high spatial reliability, enabling visualization of both the overall dispersion uniformity of MoS_2_ nanosheets within the polymer matrix and assessment of potential local enrichment or orientation. For the NF-4 membrane, the uniform distribution of nitrogen and zinc (the latter at very low content) within the PA selective layer confirmed the successful incorporation and homogeneous dispersion of ZIF-8 nanoparticles, offering key insights into the role of nanofillers in modulating the selective layer structure and separation performance. For the RO-3 membrane, the well-preserved foulant–membrane interface allowed precise characterization of foulant distribution via characteristic elements (e.g., Ca, Cl, O), facilitating a deeper understanding of membrane fouling/scaling mechanisms in actual hypersaline wastewater treatment.

Accurate characterization of membrane scaling and fouling layers is crucial for understanding their impact on system performance. The proposed sample preparation method exhibits potential advantages in membrane fouling research. Franco-Clavijo et al. highlighted the distinct morphological differences in gypsum scaling in RO membranes: growth-driven and deposition-driven scaling [38]. Goi and Liang emphasized the importance of quantifying structural parameters of foulant cakes (e.g., thickness, porosity, tortuosity) in forward osmosis (FO) for modeling the impacts of fouling, but also noted the challenges in directly measuring these properties at the nanoscale [39]. The method proposed herein can maintain the bonding state between the membrane and the scaling layer through in situ resin impregnation, avoiding structural detachment that would compromise observations of distinct scaling morphologies in the membrane. The prepared cross-sections of critical zones (e.g., RO concentration polarization zones, FO support-active layer interfaces), when combined with TEM, can capture nano-scale details, thereby clarifying mechanisms such as cake-enhanced osmotic pressure. This sample preparation method is capable of providing more reliable data for understanding scaling/fouling mechanisms and quantifying critical parameters, essential for developing accurate predictive models and effective mitigation strategies.

### 3.3. Advantages and Disadvantages Comparison with Existing Methods

Table 1 summarizes the performance differences among the three methods for preparing ultra-thin sections of polymer composite membranes: the direct resin-embedding method, cryoultramicrotomy, and the method proposed in this study. Sections prepared using the conventional direct resin-embedding method exhibited severe fragmentation, deformation, and poor thickness uniformity, attributed to significant differences in properties between the fibrous substrate and the embedding medium. This resulted in limited observable regions, as depicted in Figure 5. Moreover, this method was highly prone to sample orientation deviations, leading to inaccurate membrane thickness measurements. Although cryoultramicrotomy could achieve precise orientation using specialized fixtures [40], the weak adhesion between the sucrose embedding medium and polymer phases significantly elevated the risk of delamination within composite layers. Furthermore, both the integrity and thickness consistency of cryosections remained suboptimal due to the same underlying issues as the direct resin-embedding method.

Through the improved method proposed in this study, regions with significant disparities within the composite membrane embedding block were eliminated, thereby significantly reducing the section fragmentation rate. Simultaneously, the method enhanced resin penetration into membrane interstices, improving thickness uniformity. Delamination between membrane layers was effectively suppressed through the strong mechanical anchoring effect provided by the cured resin. Furthermore, the membrane was precisely oriented within the embedding block using the embedding device, which ensured that the membrane layers were strictly perpendicular to the sectioning direction and effectively minimized thickness measurement errors.

Despite its advantages, the developed method presents certain challenges, including a complex, multi-step protocol that requires two polymerization cycles and careful manual handling, thus limiting high-throughput application and creating a dependency on uniform resin penetration to avoid voids in dense or hydrophobic layers. However, its future scope is substantial, with potential for expansion into characterizing advanced materials like MOFs, graphene-based layers, and biological thin films. Further development could focus on commercial optimization and automation of the embedding device, integration with correlative microscopy techniques, and refinement of resin formulations to establish this method as a standardized preparation protocol for next-generation membrane and nanomaterial research.

## 4. Conclusions

This study has successfully established an innovative sample preparation protocol combining substrate stripping with a precision double-orientation embedding technique, facilitated by a purpose-designed embedding apparatus. This technique enabled the precise directional embedding of the functional layer of polymer composite membranes and preparation of high-quality ultra-thin sections. TEM analysis confirmed the elimination of conventional preparation artifacts, including section fragmentation, structural deformation, and thickness variation, which typically arise from the mechanical mismatch during cutting (density/hardness differences) and impregnation difficulties. The resulting ultra-thin sections exhibited excellent structural integrity, uniform thickness, and well-resolved membrane layer morphology. Notably, when applied to thin-film nanocomposite membranes, this preparation method enabled high-fidelity elemental distribution mapping via TEM-EDS. It successfully prevented nanoparticle displacement and region loss, ensuring reliable characterization of the distribution of nanomodified materials and fouling-layer interfaces. This technique not only provides a universal solution for composite membranes with non-woven substrates, including UF, NF, and RO membranes, but can also be extended to the cross-sectional characterization of lightweight, flexible, or ultrathin membrane materials that are difficult to orient. It offers technical support for the in situ analysis of membrane microstructure and the study of “structure-property” relationships.

## Figures and Tables

**Table 1:** The performance comparison of different methods in preparation of cross-sectional ultra-thin membrane sections.

Method	Section Integrity	Section Deformation	Thickness Uniformity	Membrane Orientation	Layer Delamination
**Direct resin-embedding method**	Low	Present	Poor	Imprecise	Low propensity
**Cryoultramicrotomy**	Low	Present	Poor	Precise	High propensity
**Method proposed in this study**	High	Absent	Good	Precise	Low propensity

“Low”: Section damage induced by the properties of the non-woven substrate in the composite membrane. “High”: The exfoliation of the non-woven substrate from the composite membrane enables the sections to remain intact.

## Data Availability

The data presented in this study are available on request from the corresponding author.

## Supplementary Materials

The following supporting information can be downloaded at: https://www.mdpi.com/article/10.3390/membranes15100288/s1, Figure S1: Elemental mappings of the cross-sectional ultra-thin sections of NF-4. (a–e) Sections prepared using the direct embedding method. (f–j) Sections prepared by the substrate stripping and double-orientation embedding technique. (b, g) C elemental maps, (c, h) N elemental maps, (d, i) O elemental maps, (e, j) Zn elemental maps. (k) EDS spectra of the NF-4 membrane cross-section confirmed the presence of zinc element from the doped ZIF-8 nanomaterials; Figure S2: Elemental mappings of the cross-sections of RO-3 membrane fouled by saline wastewater from the sewage treatment station. (a–e) Sections prepared using the direct embedding method. (f–j) Sections prepared by the substrate stripping and double-orientation embedding technique. (b, g) C elemental maps, (c, h) Ca elemental maps, (d, i) O elemental maps, (e, j) Cl elemental maps.
