# Supplementary material for: A New Method for Preparing Cross-Sections of Polymer Composite Membranes for TEM Characterization by Substrate Stripping and Double-Orientation Embedding
Source: Membranes (Basel). 2025 Sep 24;15(10):288. doi: 10.3390/membranes15100288 (PMC12566501; doi:10.3390/membranes15100288)
Supplement: Supplementary file 1 [file membranes-15-00288-s001.zip › membranes-3855078-supplementary.pdf]

Supplementary information

A new method for preparing cross-sections of polymer compo-site membranes for TEM characterization by substrate stripping and double-orientation embedding

Hongyun Ren[a,b], Zixing Zhang[a,b], Yi Li[c], Shulan Liu[a,b] and Xian Zhang[a,b,*]

[a] *Center of Analytical Instrument, Institute of Urban Environment, Chinese Academy of Sciences, Xiamen 361021, P.R. China; xzhang@iue.ac.cn*

[b] *National Key Laboratory of Regional and Urban Ecological Safety, Institute of Urban Environment, Chinese Academy of Sciences, Xiamen 361021, P.R. China*

[c] *School of Chemical Engineering and Technology, Sun Yat-sen University, Zhuhai, 519082, China; liyi266@mail.sysu.edu.cn*

* Correspondence: xzhang@iue.ac.cn

Figure S1

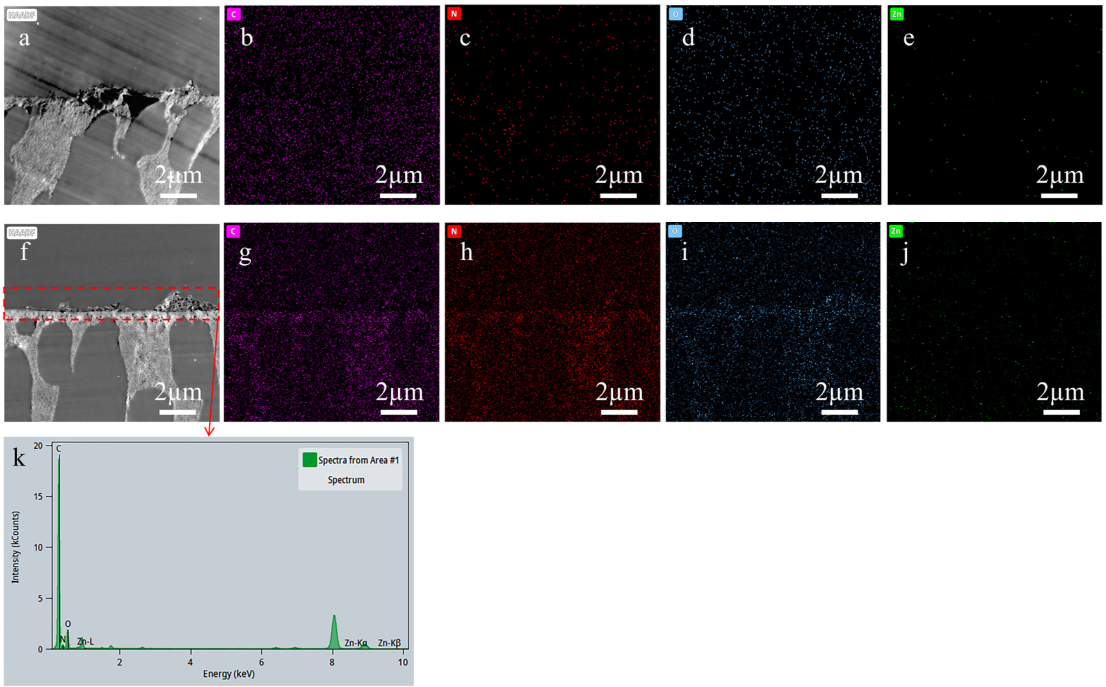

Figure S1. Elemental mappings of the cross-sectional ultra-thin sections of NF-4. (**a–e**) Sections prepared using the direct embedding method. (**f–j**) Sections prepared by the substrate stripping and double-orientation embedding technique. (**b, g**) C elemental maps, (**c, h**) N elemental maps, (**d, i**) O elemental maps, (**e, j**) Zn elemental maps. (**k**) EDS spectra of the NF-4 membrane cross-section confirmed the presence of zinc element from the doped ZIF-8 nanomaterials.

Figure S2

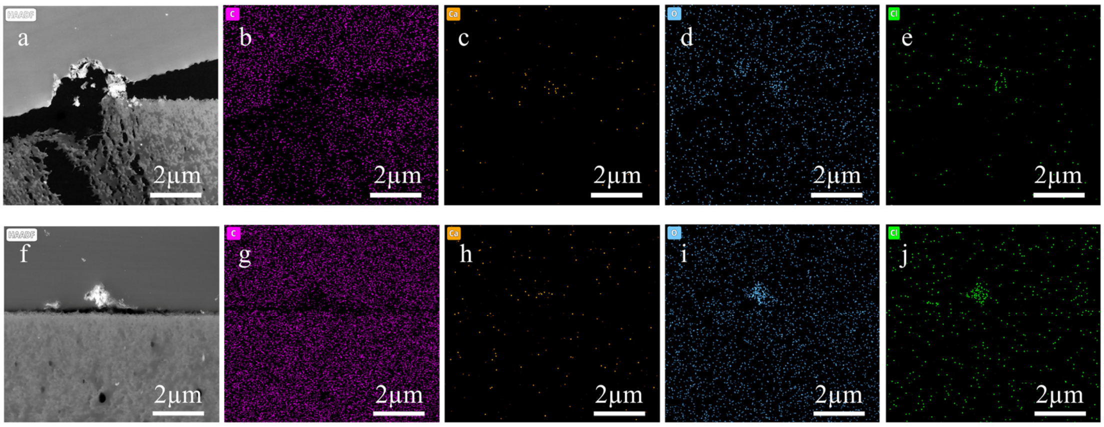

Figure S2. Elemental mappings of the cross-sections of RO-3 membrane fouled by saline wastewater from the sewage treatment plant. (**a–e**) Sections prepared using the direct embedding method. (**f–j**) Sections prepared by the substrate stripping and double-orientation embedding technique. (**b, g**) C elemental maps, (**c, h**) Ca elemental maps, (**d, i**) O elemental maps, (**e, j**) Cl elemental maps.
